# Extended Study on the Development of 3D-Printed Overlay Structures in Protective Gloves Using Ultrasonic and Contact Welding with Additional Fatigue Bending Tests

**DOI:** 10.3390/ma19040700

**Published:** 2026-02-12

**Authors:** Agnieszka Cichocka, Olga Olejnik, Emilia Irzmańska, Paulina Kropidłowska, Jakub Saramak

**Affiliations:** 1 Institute of Textiles Architecture, Faculty of Textiles and Design, Lodz University of Technology, 116 Żeromskiego Str., 90-543 Lodz, Poland; agnieszka.cichocka@p.lodz.pl; 2 Laboratory of Hand and Foot Protection, Department of Personal Protective Equipment, Central Institute for Labour Protection—National Research Institute, 16 Czerniakowska Str., 00-701 Warsaw, Poland; emirz@ciop.lodz.pl (E.I.); pakro@ciop.lodz.pl (P.K.); 3 SMK3D Company, Pabianicka 49, 95-082 Chechło Pierwsze, Poland; biuro@smk3d.pl

**Keywords:** 3D printed mesh overlay, protective materials, PPE, protective gloves, contact welding, ultrasonic welding, cyclic loading, micro-CT

## Abstract

This study investigates the development of advanced protective gloves by applying novel 3D-printed PET-G mesh overlay structures onto three textile substrates—polyamide (PA), polyester (PES), and cotton—using ultrasonic welding and contact welding. The focus was on assessing weld quality, thickness uniformity, and functional durability. Weld morphology and bonding integrity were evaluated using X-ray microtomography (micro-CT), while bending fatigue tests assessed mechanical performance under cyclic loading. The results show that ultrasonic welding produces more uniform welds, enhancing fatigue resistance, particularly on cotton and polyamide substrates. Non-uniform welds with thicker or uneven areas, typical of contact welding, correlated with reduced mechanical durability. These findings highlight the potential of additively manufactured overlay structures for hybrid protective gloves, demonstrating that weld thickness uniformity and substrate compatibility are key factors in optimizing mechanical performance. This work extends our previous research by introducing new 3D-printed overlay architectures and provides valuable insights into the practical implementation of additively manufactured polymeric structures in PPE development.

## 1. Introduction

The design and structural configuration of protective materials are fundamental to ensuring the efficiency of personal protective equipment (PPE). Depending on the specific working environment and the nature of potential hazards, materials must fulfill diverse performance requirements. Structural parameters strongly influence both the functional properties and overall effectiveness of PPE [1,2,3]. Wearer comfort is also an important factor in PPE design, as highlighted in [4] regarding underwear for foundry workers and in the detailed comparative thermal insulation analyses in [5].

The durability of, for instance, protective gloves, depends on mechanical parameters like tensile strength, tear resistance, and puncture resistance, which are especially critical for gloves used with sharp tools [6,7,8]. Protective gloves must also allow for dexterity for safe and effective manual work. Therefore, in the design process, special attention should be paid to the thickness of the material package and its structure, including the number of layers, since these parameters have a direct influence on manual dexterity and wearer comfort [9].

According to [10], the fit of PPE has a statistically significant impact on performance, reaction time, and freedom of movement. This also applies to technologies used in the production of protective gloves, such as thread and seam techniques for joining components, which can hinder work. Therefore, ergonomics is a crucial aspect of PPE design. It is reasonable to develop alternative methods to minimize or eliminate these limitations. Such solutions include stitch-free assembly using contact welding (hot plate) or ultrasonic welding, which can successfully replace traditional sewing [11,12,13,14,15,16]. These methods eliminate seam-related weaknesses, including reduced flexibility, increased thickness, and impaired air permeability. They are particularly promising for integrating 3D-printed PET-G overlays into glove structures, enabling multi-layered composites that maintain both protective performance and wearer comfort.

In the present study, we also focus on wearer-experience testing of protective gloves. This testing plays a crucial role in ensuring the effectiveness and comfort of wearers across various work environments. Protective gloves are essential equipment in industries such as manufacturing, construction, and medicine, where contact with hazardous substances, tools, or microorganisms is common. The proper selection of materials, construction, and production technologies directly affects glove functionality, durability, and wearing comfort.

A key aspect of evaluating glove quality is resistance to material fatigue, which can lead to micro-cracks, loss of protective properties, or reduced comfort. Bending fatigue tests assess glove material durability under conditions simulating real-world mechanical stress. Such tests are essential for predicting lifespan and protective efficacy [17]. For example, ref. [17] developed a method to characterize glove stiffness through multidirectional material deformations, providing a better understanding of mechanical behavior under fatigue. Another study [18] investigated the effect of wearing protective clothing and fatigue on firefighters’ functional balance during simulated operations. The results show a significant impact on balance, highlighting the importance of ergonomics and comfort in protective apparel design.

The integration of modern technologies, such as 3D printing, into protective glove production opens up new possibilities for customization, functionality, and wearer comfort. Most of the research related to 3D printing in the field of PPE focuses on the creation of novel head protection equipment, protective masks and ear protection devices [19]. However, information about preparing hand protection equipment using 3D printing technology is still lacking. Moreover, due to the pandemic, recent studies concerning the preparation of new 3D-printed PPE equipment have focused on protection against the virus itself, which remains an important topic [20,21,22]. Nevertheless, the high number of accidents involving upper limb injuries indicates that the design and manufacture of innovative protective gloves against mechanical risks is also a relevant topic [23,24,25]. In this context, Cichocka et al. [26] demonstrated that the mechanical performance of protective materials can be significantly enhanced by using basalt-based composites modified with 0.1 wt% of reduced graphene oxide (rGO). Their study highlights that the addition of rGO significantly increases cutting resistance. Furthermore, based on molecular simulations, Cichocka et al. showed that such composites form thermodynamically stable configurations and exhibit a unique crack-toughening effect due to the reformation of physical interactions at the interfaces, which prevents catastrophic failure [26]. Such advancements should be developed considering 3D printing technology application as well [7,11].

In this work, we extend the scope of investigation by introducing 3D-printed PET-G mesh overlay structures applied to PA, PES, and cotton substrates, complemented by fatigue bending evaluation. We decided to use low-cost knitted fabrics made of fibers with low mechanical resistance, such as polyamide (PA), polyester (PES) and cotton yarn. These can be enhanced using inexpensive thermoplastic overlay structures, making them competitive with high-performance textiles popular in PPE. This study continues our research on functional 3D-printed composites for protective gloves presented in [11]. Of the thermoplastic polymers tested in our previous research [11], including polylactide (PLA), poly(ethylene terephthalate)-glycol (PET-G), and acrylonitrile-butadiene-styrene (ABS), PET-G was selected to create new 3D-printed overlays integrated into protective textiles in the current study due to its lower stiffness. The chosen fused deposition modeling (FDM) method is a satisfactory 3D-printing technique used to obtain prototypes of economical tailor-made reinforcement dedicated to PPE, including protective gloves. Most papers related to FDM and knitted fabrics focus on printing thermoplastics directly onto textile carriers and only analyzing adhesion forces [27,28,29]. Furthermore, information is still scarce as regards the correlation between the quality of welds obtained in polymer-textile materials and their functional properties, including fatigue performance, as presented in the current article.

## 2. Materials and Methods

In this study, three different knitted fabrics were used: polyamide (PA), polyester (PES), and cotton (S.I. Zgoda, Konstantynów Łódzki, Poland). The samples tested are summarized in Table 1. Mesh plates as 3D-printed overlay structures were fabricated using fused deposition modeling (FDM) technology, with support from SMK3D Jakub Saramak (Chechło Pierwsze, Poland).

**Figure 1:**
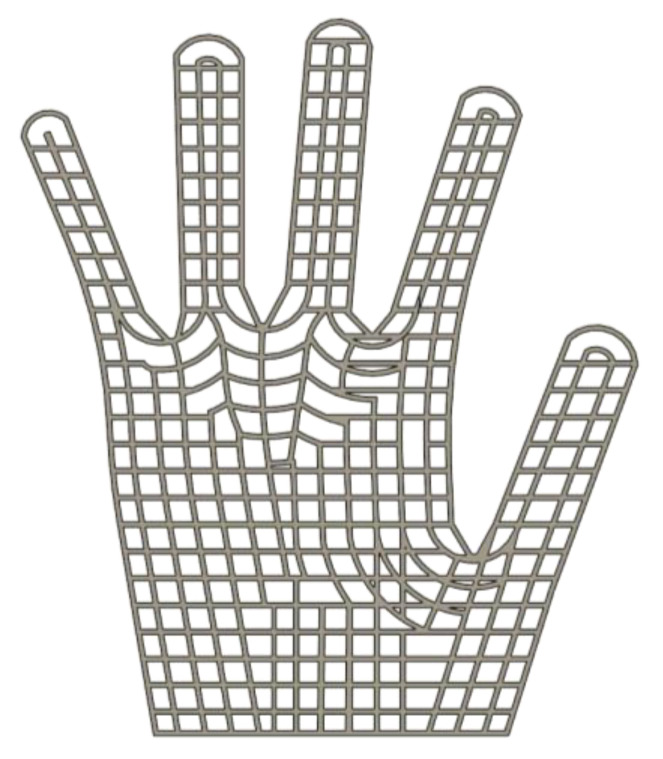
The PET-G 3D-printed structure design intended for application on protective gloves using contact welding and ultrasonic welding.

**Figure 2:**
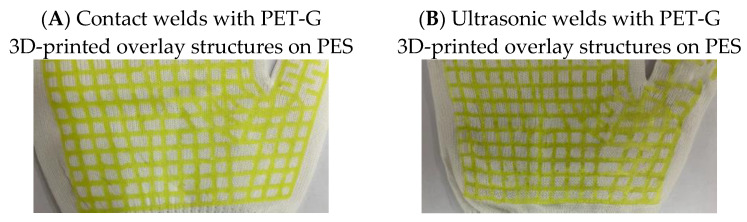
PET-G 3D-printed overlay structures applied on PES using (**A**) contact welding and (**B**) ultrasonic welding.

The 3D printing parameters were a print temperature of 245 °C and a print speed of 60 mm/s for commercially available poly(ethylene terephthalate)-glycol (PET-G) filament under the tradename Fiberlogy EASY PET-G (ρ = 1.27 g/cm^3^, Tg = 80 °C, VST = 78 °C), purchased from Fiberlab S.A. (Brzezie, Poland). The PET-G 3D-printed structures were obtained using fused deposition modeling (FDM). These structures consisted of two polymer layers, each 0.25 mm thick, produced using a 0.4 mm nozzle. The width of the lines in the mesh structure was 1.2 mm. The mesh overlay shape (Figure 1) was designed to match the glove palm with spread fingers, as illustrated in Figure 2 for PES knit fabric welded using both contact (A) and ultrasonic (B) methods.

### 2.1. Welding Methods

Optimizing ultrasonic and contact welding parameters requires considering multiple factors:

- Material type and thickness;
- Vibration frequency and amplitude;
- Welding time and pressure.

#### 2.1.1. Contact Welding Method

Contact welding (hot-plate method) was performed using a multifunctional digital heat transfer press (TLM13135, VEVOR, Yiwu, China) with a PTFE-covered platen. The procedure included:

▪ Placing the knitted fabric (PA, PES, or cotton) on a stationary table.
▪ Positioning the PET-G overlay structure on the fabric.
▪ Covering the assembly with baking paper to prevent contamination.
▪ Applying the heated platen and pressing with a clamp for 35 s.
▪ Adjusting the temperature by substrate: 160 °C for cotton, PA and PES.

#### 2.1.2. Ultrasonic Welding Method

Ultrasonic welding was applied to thermoplastic substrates (PA and PES) and extended to cotton. Previous studies [12] explored embedding optical fibers in cotton fabrics via ultrasonic welding, analyzing heating and bonding mechanisms and the effects of weld pressure, time, and vibration amplitude. The results showed that ultrasonic welding can integrate fibers while maintaining joint strength and signal stability.

Attention was given to bonding the 3D mesh overlay to cotton knit fabric. Despite the absence of inherent thermoplastic behavior, stable and reproducible joints were achieved by adjusting vibration frequency and amplitude. Parameter optimization followed an iterative experimental approach, allowing the identification of a processing window where frictional heating and mechanical interlocking produced reliable interfaces. However, the final parameters were as follows: 1.7–2.5 m/min of advance speed and 0.2–1 bar of pressure for a 5 mm weld width.

### 2.2. X-Ray Microtomography (Micro-CT)

High-resolution X-ray computed tomography (micro-CT) (Bruker, Kontich, Belgium) was used to assess the contact area between mesh overlay structures made of PET-G and selected knitted fabrics, including polyester (PES), polyamide (PA) and cotton (CO). Scans were performed according to [11] using a SkyScan 1272 system (Bruker, Kontich, Belgium) at 50 kV and 200 µA, with a pixel size of 5.5 µm, over a 180° rotation with a 0.2° step, with no filter. The quantitative analysis was carried out using CTAn (v. 1.14.4).

### 2.3. Bending Fatigue Test

To validate the conclusions based on micro-CT data, we decided to confront them with performance tests used in protective materials evaluation. For this purpose, we conducted fatigue bending tests on the finished gloves. This approach was methodologically justified, as laboratory results must be validated under conditions that simulate real-world use.

The bending fatigue tests were conducted using a De Mattia apparatus, adapting the procedure from the PN-EN-ISO 7854:2002 standard [30]. The adaptation was necessary since there are no existing standards that fully address the specific nature of the tested samples—substrates (knitted fabrics) with attached perforated plates—as opposed to fully coated gloves, to which the norm applies. However, the adjusted procedure of the bending fatigue test was as follows: First, each narrow strip of fabric reinforced with mesh overlay structures was folded twice (with the edges together) to form a small rectangular package. This folded sample was then clamped between two flat grips on a flexing machine. One of the grips moved up and down at a frequency of about 5 Hz, repeatedly bending the sample outwards. The test was carried out for 100 cycles. Then, the sample was observed, after which the test continued until visible damage was first detected without a microscope. Nevertheless, when the crack was visible to the naked eye, then photos of the cracked sample were taken using a SUNSHINE digital microscope (Guangzhou Sunshine Electronic Technology Co., Ltd., Guangzhou, China) with 7× magnification.

The fatigue tests were designed to make a first approach to examine whether the measured contact surface area, as well as the development surface, determined by micro-CT, translates into increased resistance to cracking and delamination under cyclical stress.

The evaluation for cracking on the surface of the plate was performed every 100 cycles using a simplified scale: 0 meaning no visible changes and 1 meaning cracks present. To determine the characteristics of crack depth with evaluation, the following indicators were used:

- 0—no cracks;
- A—cracks on the surface or in the top layer, without delamination from the substrate.

These tests are a key element of the evaluation, as they allow for a holistic verification of mesh overlay joint quality, combining morphological data with actual mechanical performance. The conclusion, based on the full spectrum of tests, will allow for a precise selection of the optimal joining technology depending on the specific product requirements.

## 3. Results

### 3.1. Ultrasonic and Contact Welding on X-Ray Microtomography (Micro-CT)

In our study, we evaluate the quality of welded joints obtained in textiles, in materials using micro-CT, like the research in [31,32].

#### 3.1.1. Analysis of Surface Contact Area and Surface Development of Welds

Table 2 presents the comparison and contact and surface development parameters of the tested samples. The largest contact area was obtained in the PA_C system (48.72 mm^2^), which indicates a very good bond between the overlay and the PA substrate. The smallest values for the contact area were recorded for CO_U (10.95 mm^2^). PES showed intermediate values (20–27 mm^2^), with clearly better contact parameters achieved using the contact welding method. The highest surface development was observed for PA_C (903%), indicating the greatest degree of mechanical anchoring of the coating in the knit structure. The lowest development was recorded for PES_U (428%), while higher surface development was observed for both cotton and polyester knits.

Figure S1 presents a combined bar and line chart showing two key parameters of weld quality: contact area (bars) and surface development (line). The six tested variants are based on three types of knitted fabric substrates (PA, CO, and PES) and two welding methods (C—contact welding; U—ultrasonic welding).

Overall, the values obtained with the ultrasonic method were lower, but their variation was significant.

Notably, ultrasonic welding on polyamide (PA_U) yielded considerably better results (contact area ~37 mm^2^ and surface development ~660%) compared to other knits (CO_U, PES_U).

#### 3.1.2. Analysis of Weld Thickness Distributions

**Figure 3:**
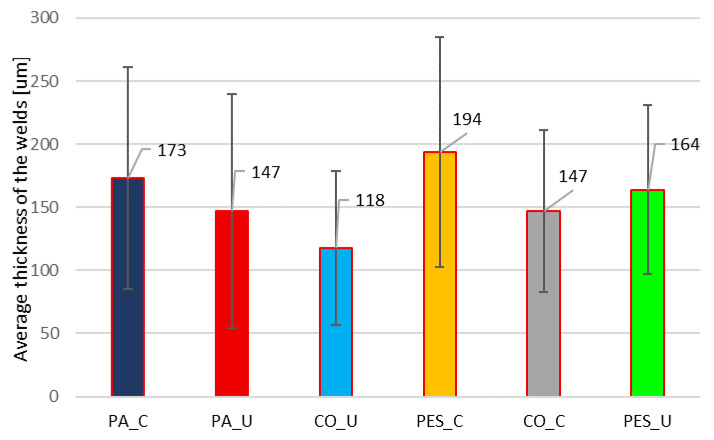
The average thickness and uniformity of the welds (standard deviation—SD) for six variants of tested samples.

The welds obtained using two joining methods, contact (C) and ultrasonic (U) welding, were analyzed taking into account weld thickness distribution and applying the microtomographic (micro-CT) method. The results received are presented in Table S1. The following analysis focuses on the average thickness and uniformity of the welds (standard deviation—SD), which are presented in Figure 3.

Each row in Table S1 corresponds to a weld morphology oriented to a thickness range in micrometers (µm), e.g., 5–15 µm, 15–25 µm, etc. That “Midpoint” is the average value for a given interval, used for creating distribution charts. Each column for a weld (cotton, PA, PES, etc.) shows the percentage of the surface area that falls within a specific thickness range. For example, for the PA_U welds, 5.6083% of the total analyzed area is within the 15–25 µm range.

The proposed analysis shows the uniformity of the applied layer and how that is important but also how much the thickness varies in different places. Additionally, it is possible to identify defects or thinner/thicker areas of the welds, especially since if the welds are too thin in certain areas, it can affect mechanical strength, chemical resistance, or barrier properties. For example, the CO_U welds have a mean thickness of 118 µm and a standard deviation of 61 µm (relatively uniform), while the PES_C has 194 µm and a standard deviation of 91 µm (more uneven).

**Figure 4:**
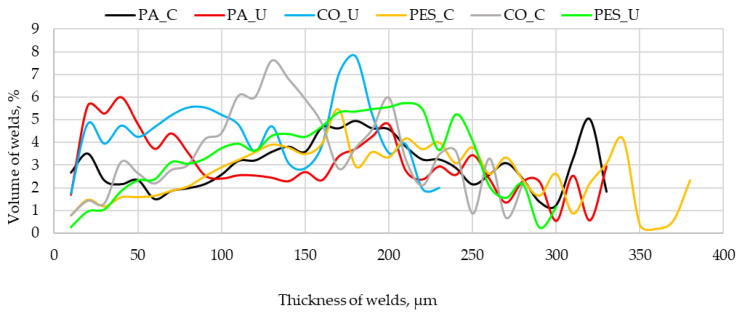
Volumetric thickness distributions of coatings for six variants of tested samples.

Figure 4 presents the line graph on the volumetric thickness distributions of welds for six variants obtained by contact (C) and ultrasonic welding (U). Each line represents a unique thickness profile for a given material system. The lines for the ultrasonic method (red—PA_U, blue—CO_U, and green—PES_U) are characterized by narrower, more concentrated peaks. This indicates that most of the weld material is found within a relatively narrow thickness range, which translates to greater uniformity. The most uniform profile was observed for PA_U, whose line shows the highest and sharpest peak compared to the other ultrasonic methods. The contact method (C) creates welds that are thicker but less uniform because the lines for the contact method (black—PA_C, yellow—PES_C, gray—CO_C) are wider and more spread out. In addition to distinct peaks, these lines also have “tails” that extend deeper into the graph toward higher thicknesses (above 300 µm). This indicates a greater spread in thickness and the presence of areas with accumulated weld materials.

When comparing the methods, the thickness distributions are unique for each variant. For example, although both ultrasonic methods on the substrates polyamide (PA_U) and cotton (CO_U) create uniform welds, the thickness profile for PA_U is more compact. For that reason, the influence of the knit fabric (substrate) type on the weld thickness distribution is crucial.

### 3.2. Bending Fatigue Test

Bending fatigue tests were conducted on substrates with 3D-printed overlays using the De Mattia method, adapting the procedure from the PN-EN-ISO 7854:2002 standard. The aim was to determine whether the contact surface area and surface development measured by micro-CT correlate with resistance to cracking and delamination under cyclic loading. Cracks were evaluated every 100 cycles using a simplified 0–1 scale.

The bending fatigue test (Table 3) shows that the cotton substrate with ultrasonic welding (CO_U) exhibited the highest resistance to cracking, with 800 cycles without cracks. The polyamide substrates performed moderately with PA_U for 400 cycles and PA_C for 300 cycles. The polyester substrates showed the lowest fatigue resistance with PES_C for 200 cycles and PES_U for 300 cycles. In general, ultrasonic welding improved fatigue performance compared to contact welding, and the substrate type strongly influenced the number of cycles without cracks.

## 4. Discussion

Our research on the uniformity of weld thickness, measured by X-ray microtomography (micro-CT), proves that this is a key factor influencing bending fatigue resistance. We observed that ultrasonic welding improves fatigue performance compared to the contact method, which is due to the creation of more uniform welds. Ultrasonic welds are characterized by narrower, more concentrated thickness peaks, while contact welding leads to the formation of thicker, less uniform welds with broader peaks and extended tails.

This difference is reflected in the results of the fatigue tests, where ultrasonic welds on PA and CO substrates (PA_U: 400 cycles, CO_U: 800 cycles) outperformed contact welds (PA_C: 300, CO_C: 600 cycles).

The type of substrate plays a crucial role in weld quality and durability. Cotton (CO) provides the most favorable weld characteristics and the highest fatigue resistance, especially with the use of ultrasonic welding, which correlates with a uniform thickness profile. In contrast, polyamide (PA) shows a moderate improvement with ultrasonic welding, and polyester (PES) shows the lowest resistance, presumably due to a less favorable thickness distribution and weld irregularities. Compared to synthetic fibers, cotton generally exhibits weaker bonding with thermoplastic polymers such as PET-G. This is mainly due to the relatively smooth surface morphology of cotton fibers and their chemical affinity towards most thermoplastics [33,34]. While a better chemical affinity between a thermoplastic polymer and a textile fabric (for instance, PET-G and polyester knit fabric) may improve adhesion, it may also negatively affect functional properties such as fatigue performance due to limited movement. If the thermoplastic layer is bonded too strongly to the knitted substrate, it may lead to premature cohesive failure of the polymer, as stress relaxation mechanisms at the interface are suppressed [35].

There is also a clear correlation between the uniformity of weld thickness and fatigue resistance. Narrow, concentrated thickness peaks in ultrasonic welds increase durability, while wider and more uneven welds from the contact method lower fatigue performance, supposedly due to localized stress concentration. Samples with uniform thickness profiles (ultrasonic welds) can withstand more bending cycles without cracking.

A quantitative analysis of the micro-CT data in relation to the bending fatigue tests reveals an inverse correlation between the contact surface area and fatigue life. For instance, the PA_C samples exhibited the highest contact surface area (48.72 mm^2^) and surface development (903%), indicating a very strong adhesive bond. However, this resulted in only 300 fatigue cycles without cracking. In contrast, the CO_U samples, which showed the lowest contact surface area (10.95 mm^2^), demonstrated the highest fatigue resistance, withstanding up to 800 cycles. This suggests that an excessively large contact area creates a stiff interface that prevents micro-movements between the overlay and the substrate, leading to stress concentration and premature failure. The ultrasonic welding method on cotton (CO_U) produces a specific connection where the bonding is sufficient to hold the structure but “loose” enough (lower contact area) to allow for stress dissipation during cyclic bending. Furthermore, the PES_C samples, characterized by a high standard deviation in weld thickness (91 µm) and a broad thickness distribution profile, failed the fastest (200 cycles), confirming that lack of uniformity directly accelerates crack initiation.

Although weld uniformity is important when comparing the same material prepared via different welding methods, the type of substrate plays a more decisive role, as can be seen in the results of the fatigue tests. When implementing PET-G reinforcement on knitted fabric to prevent premature cracking a weaker interaction between the thermoplastic and textile carrier is required. Therefore, a cotton textile carrier is the most suitable to obtain more a fatigue-resistant hybrid material dedicated to protective gloves.

## 5. Conclusions

The results obtained, although representing initial trials in this field, and additionally complemented by bending fatigue tests, prove that ultrasonic welding technology can be successfully adapted to multi-material assemblies of protective gloves, including those with substrates lacking intrinsic thermoplastic properties. The potential of this method extends beyond conventional polymer textiles, enabling the creation of hybrid systems with natural fibers. Through parameter optimization, this technology opens up new possibilities in the design of protective gloves reinforced with 3D-printed overlays. High-resolution X-ray computed tomography (micro-CT) can be used to assess the contact area between novel 3D-printed thermoplastic structures and textiles, particularly the uniformity of weld thickness carriers, as this affects fatigue resistance. The micro-CT analysis method is helpful during comparisons of the same type of material prepared using various welding methods and conditions. However, the type of substrate, including the textile carrier, is more relevant, as chemical affinity plays an important role in functional properties, which is also noticeable in fatigue test results. Our findings suggest that further research and a systematic evaluation of process parameters can solidify this approach and confirm its relevance to broader industrial applications.

## Figures and Tables

**Table 1:** Knit fabric parameters (S.I. Zgoda, Konstantynów Łódzki, Poland).

Knit Fabric Description	Knit Fabric Weight [g/m^2^]	Weave
PA	231.9	Jersey (resultant yarn count: 50.01 tex (3 ends of 75 den/2))
PES	222.1	Jersey (resultant yarn count: 50.01 tex (3 ends of 75 den/2))
Cotton	201.8	Jersey (resultant yarn count: 50.01 tex (3 ends of 75 den/2))

**Table 2:** Comparison of contact surface area and surface development of the tested samples from micro-CT.

Substrates_Welds [C—Contact] [U—Ultrasonic]	Contact Surface Area of Welds Substrates [mm^2^]	Surface Development [%]
PA_C	48.72	903
PA_U	36.87	662
CO_U	10.95	627
CO_C	27.54	696
PES_C	26.90	539
PES_U	20.27	428

**Table 3:** Evaluation of bending fatigue test via Digital Microscope SUNSHINE 7× magnification.

Sample of Knit Fabric	3D-Printed Overlay Structures Welds on Knit Fabric Before Bending Fatigue Test	3D-Printed Overlay Structures Welds on Knit Fabric After Bending Fatigue Test	Number of Cycles with No Cracks
PA_C	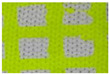	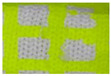	300
PA_U	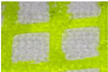	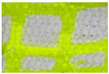	400
CO_U	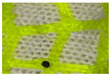	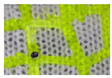	800
CO_C	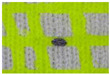	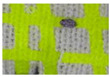	600
PES_C	** 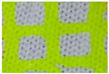 **	** 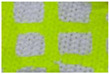 **	200
PES_U	** 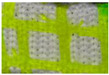 **	** 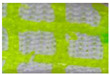 **	300

## Data Availability

The original contributions presented in this study are included in the article/Supplementary Materials. Further inquiries can be directed to the corresponding author.

## Supplementary Materials

The following supporting information can be downloaded at: https://www.mdpi.com/article/10.3390/ma19040700/s1, Table S1: Thickness distribution of welds; Figure S1: Comparison of contact area and surface development based on the welding method and knit fabric type.

## References

[1] Dolez P.I., Marsha S., McQueen R.H. (2022). Fibers and Textiles for Personal Protective Equipment: Review of Recent Progress and Perspectives on Future Developments. Textiles.

[2] Shi J., Li H., Xu F., Tao X. (2021). Materials in Advanced Design of Personal Protective Equipment: A Review. Mater. Today Adv..

[3] Dolez P.I., Vu-Khanh T. (2009). Recent Developments and Needs in Materials Used for Personal Protective Equipment and Their Testing. Int. J. Occup. Saf. Ergon..

[4] Gilewicz P., Cichocka A., Frydrych I. (2016). Underwear for Protective Clothing Used by Foundry Workers. Fibres Text. East. Eur..

[5] Frydrych I., Cichocka A., Adamczyk P., Dominiak J. (2016). Comparative Analysis of the Thermal Insulation of Traditional and Newly Designed Protective Clothing for Foundry Workers. Polymers.

[6] Kropidłowska P., Jurczyk-Kowalska M., Irzmańska E., Płociński T., Laskowski R. (2021). Effects of Composite Coatings Functionalized with Material Additives Applied on Textile Materials for Cut Resistant Protective Gloves. Materials.

[7] Żyłka E., Irzmańska E., Saramak J., Jurczyk-Kowalska M. (2024). Functional 3D-Printed Polymeric Materials with Metallic Reinforcement for Use in Cut-Resistant Gloves. Materials.

[8] Kropidłowska P., Irzmańska E., Sawicki J. (2022). Preliminary Experimental Investigation of Cut-Resistant Materials: A Biomimetic Perspective. Autex Res. J..

[9] Karim N., Afroj S., Lloyd K., Oaten L.C., Andreeva D.V., Carr C., Farmery A.D., Kim I.D., Novoselov K.S. (2020). Sustainable Personal Protective Clothing for Healthcare Applications: A Review. ACS Nano.

[10] Brisbine B.R., Radcliffe C.R., Jones M.L.H., Stirling L., Coltman C.E. (2022). Does the Fit of Personal Protective Equipment Affect Functional Performance? A Systematic Review across Occupational Domains. PLoS ONE.

[11] Irzmańska E., Cichocka A., Puszkarz A.K., Olejnik O., Kropidłowska P. (2024). A New Approach to Implementing 3D-Printed Material Structures for Protective Gloves with the Use of Ultrasonic and Contact Welding Processes: A Preliminary Study. Materials.

[12] Shi W., Little T. (2000). Mechanisms of ultrasonic joining of textile materials Available to Purchase. Int. J. Cloth. Sci. Technol..

[13] Yıldız E.Z. (2023). The Effect of Fabric Structure and Ultrasonic Welding Process on the Performance of the Spunlace Surgical Gowns. Tekst. Konfeksiyon.

[14] Jevšnik S., Eryürük S.H., Kalaoğlu F., Kayaoğlu B.K., Komarkova P., Golombikova V., Stjepanovič Z. (2017). Seam Properties of Ultrasonic Welded Multilayered Textile Materials. J. Ind. Text..

[15] Quintino L. (2013). Introduction to Joining Methods in Medical Applications. Joining and Assembly of Medical Materials and Devices.

[16] Qiu J., Zhang G., Sakai E., Liu W., Zang L. (2020). Thermal Welding by the Third Phase between Polymers: A Review for Ultrasonic-weld Technology Developments. Polymers.

[17] Harrabi L., Dolez P.I., Vu-Khanh T., Lara J., Tremblay G., Nadeau S., Larivière C. (2008). Characterization of protective gloves stiffness: Development of a multidirectional deformation test method. Saf. Sci..

[18] Hur P., Rosengren K.S., Horn G.P., Smith D.L., Hsiao-Wecksler E.T. (2013). Effect of Protective Clothing and Fatigue on Functional Balance of Firefighters, Human Performance and Comfort in Protective Clothing and Sportswear. J. Ergon..

[19] Jafferson J.M., Pattanashetti S. (2021). Use of 3D printing in production of personal protective equipment (PPE)—A review. Mater. Today Proc..

[20] Bharti N., Singh S. (2020). COVID-19: The Use of 3D Printing to Address PPE Short age during a Pandemic—A Safety Perspective. ACS Chem. Health Saf..

[21] Luchini K., Sloan S.N.B., Mauro R., Sargsyan A., Newman A., Persaud P., Hawkins D., Wolff D., Staudinger J., Creamer B.A. (2021). Sterilization and sanitizing of 3D-printed personal protective equipment using polypropylene and a Single Wall design. 3D Print. Med..

[22] Agarwal R. (2022). The personal protective equipment fabricated via 3D printing technology during COVID-19. Ann. 3D Print. Med..

[23] Khanlari P., Ghasemi F., Heidarimoghdam R. (2023). Protective gloves, hand grip strength, and dexterity tests: A comprehensive study. Heliyon.

[24] Sorock G.S., Lombardi D.A., Peng D.K., Hauser R., Eisen E.A., Herrick R.F., Mittleman M.A. (2010). Glove Use and the Relative Risk of Acute Hand Injury: A Case-Crossover Study. J. Occup. Environ. Hyg..

[25] Lim J., Cho J., Kim J., Kang S. (2024). Workers’ Injury Risks Focusing on Body Parts in Reinforced Concrete Construction Projects. Int. J. Environ. Res. Public Health.

[26] Cichocka A., Frydrych I., Zawadzki P., Kaczmarek Ł., Irzmanska E., Kropidłowska P. (2025). Basalt-Based Composite with Reduced Graphene Oxide (rGO)—Preliminary Study on Anti-Cut Properties. Materials.

[27] Ehrmann A. (2023). Adhesion of New Thermoplastic Materials Printed on Textile Fabrics. Tekstilec.

[28] Han Y., Yun C. (2024). Effect of substrate fabric characteristics on the peel strength of 3D-printed composite fabrics. Fash. Text..

[29] Silvestre R., Garcia-Breijo E., Ferri J., Montava I., Bou-Belda E. (2023). The Influence of the Structure of Cotton Fabrics on the Adhesion of Conductive Polymer Printed with 3D Printing Technology. Polymers.

[30] (2002). Rubber- or Plastics-Coated Fabrics—Determination of Resistance to Damage by Flexing.

[31] Renard M., Machnowski W., Puszkarz A.K. (2023). Assessment of Thermal Performance of Phase-Change Material-Based Multilayer Protective Clothing Exposed to Contact and Radiant Heat. Appl. Sci..

[32] Pabjańczyk-Wlazło E.K., Puszkarz A.K., Bednarowicz A., Tarzyńska N., Sztajnowski S. (2022). The Influence of Surface Modification with Biopolymers on the Structure of Melt-Blown and Spun-Bonded Poly(Lactic Acid) Nonwovens. Materials.

[33] Wankhede B., Bisaria H., Ojha S., Dakre V.S. (2022). A review on cotton fibre-reinforced polymer composites and their applications. Proc. Inst. Mech. Eng. Part L J. Mater. Des. Appl..

[34] Zhou Y., Fan M., Chen L. (2016). Interface and bonding mechanisms of plant fibre composites: An overview. Compos. Part B.

[35] Li M., Mao A., Guan Q., Saiz E. (2024). Nature-inspired adhesive systems. Chem. Soc. Rev..

