# Supplementary material for: Extended Study on the Development of 3D-Printed Overlay Structures in Protective Gloves Using Ultrasonic and Contact Welding with Additional Fatigue Bending Tests
Source: Materials (Basel). 2026 Feb 12;19(4):700. doi: 10.3390/ma19040700 (PMC12942377; doi:10.3390/ma19040700)
Supplement: Supplementary file 1 [file materials-19-00700-s001.zip › materials-4006934-supplementary.pdf]

materials

MDPI

Supplementary Material

Extended study on the development of 3D-printed overlay structures in protective gloves using ultrasonic and contact welding with additional fatigue bending tests

Agnieszka Cichocka [1], Olga Olejnik [2]*, Emilia Irzmańska [2], Paulina Kropidłowska [2], Jakub Saramak[3]

1. Lodz University of Technology, Faculty of Material Technologies and Textile Design, Institute of Textiles Ar-chitecture, 116 Żeromskiego Str., 90-543 Lodz, Poland; agnieszka.cichocka@p.lodz.pl
2. Central Institute for Labour Protection – National Research Institute, Department of Personal Protective Equipment, Wierzbowa 48, 90-133 Lodz, Poland; olgol@ciop.lodz.pl, emirz@ciop.lodz.pl, pakro@ciop.lodz.pl
3. SMK3D Company, 49 Pabianicka., 95-082 Chechło Pierwsze, Poland; biuro@smk3d.pl

* Correspondence: olgol@ciop.lodz.pl; tel.: +48 42 648 02 46

Table S1. Thickness distribution of welds

Thickness of welds							
Range	Midpoint	PA_C	PA_U	CO_U	PES_C	CO_C	PES_U
µm	µm	%	%	%	%	%	%
5-15	10	2,6703	1,6985	1,752	0,7886	0,8	0,2566
15-25	20	3,5045	5,6083	4,8235	1,4785	1,4443	0,9461
25-35	30	2,3218	5,2641	3,9448	1,1845	1,3308	1,0419
35-45	40	2,1576	5,9901	4,7409	1,5891	3,1571	1,8437
45-55	50	2,3401	4,8075	4,2463	1,5941	2,6538	2,3165
55-65	60	1,5078	3,7185	4,6756	1,6535	2,2047	2,3622
65-75	70	1,8678	4,3948	5,1996	1,8726	2,7992	3,1221
75-85	80	1,984	3,5422	5,5393	2,0643	3,0264	3,0532
85-95	90	2,1648	2,5495	5,54	2,5106	4,1556	3,2704
95-105	100	2,5801	2,407	5,2118	2,9105	4,4309	3,7366
105-115	110	3,1823	2,5539	4,7838	3,226	6,0651	3,9332
115-125	120	3,2058	2,5441	3,5981	3,569	6,0107	3,6254
125-135	130	3,5802	2,447	4,7106	3,904	7,6162	4,2804
135-145	140	3,8087	2,2957	3,0829	3,7738	6,7996	4,3585
145-155	150	3,5857	2,7016	2,8681	3,4922	5,9074	4,2366
155-165	160	4,6553	2,3389	3,8804	3,9466	4,8175	4,6967
165-175	170	4,6098	3,3829	7,0279	5,4588	2,8494	5,3081
175-185	180	4,939	3,7145	7,794	2,9609	3,7722	5,3485
185-195	190	4,6134	4,2594	5,2033	3,5826	4,5921	5,4602
195-205	200	4,5683	4,7867	3,5281	3,3487	5,9743	5,5489
205-215	210	3,8177	2,7614	3,9103	4,1757	3,265	5,7247
215-225	220	3,2454	2,3679	1,9422	3,7026	2,1191	5,4512
225-235	230	3,2472	2,944	1,9966	4,001	3,4835	3,6458
235-245	240	2,8747	2,5712		3,0906	3,6137	5,2255
245-255	250	2,1491	3,4411		3,7848	0,8762	4,0509
255-265	260	2,577	2,3991		2,6175	3,2982	2,0633

265-275	270	3,0889	1,3516		3,3396	0,6838	1,5489
275-285	280	2,3886	2,2924		2,3566	2,2531	2,1498
285-295	290	1,3832	2,2677		1,6591		0,2344
295-305	300	1,246	0,5475		2,6209		1,1597
305-315	310	3,2831	2,537		0,8733		
315-325	320	5,0187	0,57		2,189		
325-335	330	1,8333	2,9441		3,0685		
335-345	340				4,1393		
345-355	350				0,3763		
355-365	360				0,2024		
365-375	370				0,5566		
375-385	380				2,3374		
**Average**		**173**	**147**	**118**	**194**	**147**	**164**
**SD**		**88**	**93**	**61**	**91**	**64**	**67**

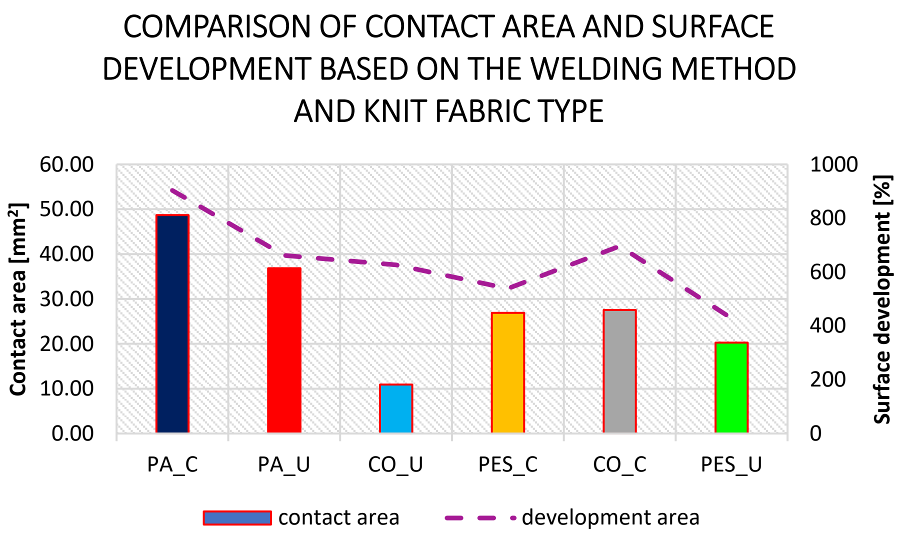

Figure S1. Comparison of contact area and surface development based on the welding method and knit fabric type
